# Prolyl 3-Hydroxylase 2 Is a Molecular Player of Angiogenesis

**DOI:** 10.3390/ijms22083896

**Published:** 2021-04-09

**Authors:** Paola Pignata, Ivana Apicella, Valeria Cicatiello, Caterina Puglisi, Sara Magliacane Trotta, Remo Sanges, Valeria Tarallo, Sandro De Falco

**Affiliations:** 1 Institute of Genetics and Biophysics ‘Adriano Buzzati-Traverso’—CNR, Angiogenesis LAB, 80131 Naples, Italy; paola.pignata@igb.cnr.it (P.P.); apicella.ivana@gmail.com (I.A.); valeria.cicatiello@igb.cnr.it (V.C.); sara.magliacanetrotta@igb.cnr.it (S.M.T.); valeria.tarallo@igb.cnr.it (V.T.); 2 IOM Ricerca s.r.l., 95029 Viagrande, Italy; caterina.puglisi@grupposamed.com; 3 Computational Genomics Laboratory, International School for Advanced Studies (SISSA), 34136 Trieste, Italy; remo.sanges@gmail.com; 4 ANBITION s.r.l., Department of R&D, 80128 Napoli, Italy

**Keywords:** angiogenesis, prolyl 3-hydroxylase 2, Collagen IV, vascular endothelial growth factor A, choroidal neovascularization, age-related macular degeneration

## Abstract

Prolyl 3-hydroxylase 2 (*P3H2*) catalyzes the post-translational formation of 3-hydroxyproline on collagens, mainly on type IV. Its activity has never been directly associated to angiogenesis. Here, we identified *P3H2* gene through a deep-sequencing transcriptome analysis of human umbilical vein endothelial cells (HUVECs) stimulated with vascular endothelial growth factor A (VEGF-A). Differently from many previous studies we carried out the stimulation not on starved HUVECs, but on cells grown to maintain the best condition for their in vitro survival and propagation. We showed that *P3H2* is induced by VEGF-A in two primary human endothelial cell lines and that its transcription is modulated by VEGF-A/VEGF receptor 2 (VEGFR-2) signaling pathway through p38 mitogen-activated protein kinase (MAPK). Then, we demonstrated that *P3H2*, through its activity on type IV Collagen, is essential for angiogenesis properties of endothelial cells in vitro by performing experiments of gain- and loss-of-function. Immunofluorescence studies showed that the overexpression of *P3H2* induced a more condensed status of Collagen IV, accompanied by an alignment of the cells along the Collagen IV bundles, so towards an evident pro-angiogenic status. Finally, we found that *P3H2* knockdown prevents pathological angiogenesis in vivo, in the model of laser-induced choroid neovascularization. Together these findings reveal that *P3H2* is a new molecular player involved in new vessels formation and could be considered as a potential target for anti-angiogenesis therapy.

## 1. Introduction

Vascular Endothelial Growth Factor A (VEGF-A) is the most potent stimulator of new blood vessels formation from pre-existing ones, a dynamic and complex process known as angiogenesis [1]. Due to the central role of VEGF-A and related tyrosine kinase receptors, VEGF receptor 1 (VEGFR-1) and VEGF receptor 2 (VEGFR-2) [2], initial efforts in the search for anti-angiogenic therapeutic agents have been focused on molecules able to neutralize their activity. Despite the development of several agents directed against alternative therapeutic targets, until now the anti-VEGF therapy represents the main performing approach in anti-angiogenic therapy [3,4].

Although anti-VEGF therapy is clinically helpful, as demonstrated by improved survival in cancer patients or visual acuity in age-related macular degeneration (AMD) patients [3,5], many of them are refractory to this therapy or develop severe side effects [6,7,8]. Consequently, the search of new angiogenesis players is highly demanded in order to explore alternative and more safe therapeutic approaches.

In this perspective, we performed a transcriptome (RNAseq) analysis on human umbilical vein endothelial cells (HUVECs) stimulated with recombinant VEGF-A. Differently from previous studies conducted with the same actors [9,10,11,12,13,14,15], we decided to carry out the stimulation not on starved HUVECs, but on cells grown to maintain the best condition for their in vitro survival and propagation. The RNAseq analysis highlighted 459 differentially expressed genes. Of note, the peculiar conditions chosen for the stimulation of HUVECs allowed us also to identify some genes whose function has never been correlated with the activity of VEGF-A and more in general with angiogenesis process so far. Among these genes we have focused our attention on prolyl 3-hydroxylase 2 (*P3H2*).

*P3H2* [16], also known as Leucine proline-enriched proteoglycan (Leprecan) like 1 (LEPREL1) [17], belongs to the family of prolyl 3-hydroxylases (P3H1, *P3H2*, and P3H3) enzymes, which are involved in the post-translational modification of collagens [18,19]. Differently from P3H1, which has been previously identified as a basement membrane-associated glycoprotein in rats (LEPRECAN) [20] and then as a potential growth suppressor (growth suppressor 1, Gros1) gene in mice [21], *P3H2* is localized to the endoplasmic reticulum and Golgi network and it is mainly expressed in human placenta, lung, liver, heart, and kidney [16,17].

P3Hs catalyze the hydroxylation of Pro in 3-hydroxyl-l-proline (3Hyp) in position Xaa of the repeating Xaa-Yaa-Gly triplets characteristic of collagen sequence. Differently from prolyl 4-hydroxylases (C-P4Hs) that catalyze the hydroxylation of Pro in 4-hydroxyl-l-proline (4Hyp) in position Yaa, with a frequency of around 100 residues per 1000 amino acids similar in different collagen types, the modifying activity of P3Hs markedly varies between different collagens. Indeed, P3H1 acts primarily on type I collagen modifying 1 residue of Pro every 1000 amino acids. *P3H2* shows a similar activity on Collagen I but its main substrate is represented by Collagen type IV, with the modification of 10–15 Pro residues every 1000 amino acids [22,23].

Collagen IV is the main component of the basement membrane and it has been associated to the angiogenic process. It is able to modulate some functions of endothelial cells directly related to the angiogenesis process, such as cell adhesion and migration [24,25], proliferation [26], and morphological differentiation [27,28]. In in vitro model of angiogenesis such as capillary sprouting from aortic ring, Collagen IV accumulates gradually in the subendothelial space, appearing as a patchy subendothelial deposit in the early stages of angiogenesis, whereas in the late stages it forms a continuous feltwork around the newly formed microvessels [29]. When aortic rings were cultured in the presence of Collagen IV, neovessels elongation, and survival were promoted in a dose-dependent manner and high concentration of Collagen IV were able to stabilize the neovascular outgrowths preventing vascular regression [30].

Here, we report the results and the validation of the deep-sequencing transcriptome analysis of HUVECs stimulated with recombinant VEGF-A. We confirm that *P3H2* expression is induced by VEGF-A in two primary human endothelial cell lines, HUVECs and the human dermal microvascular endothelial cells (HDMVECs). We have investigated which signaling pathway is involved and we have performed gain- and loss-of-function experiments to study the impact of the modulation of *P3H2* expression on angiogenic properties of primary endothelial cells and on Collagen IV. Finally, we have examined the consequence of *P3H2* knockdown in vivo in the model of laser-induced choroid neovascularization (CNV).

## 2. Results

### 2.1. Identification of New Genes Modulated by VEGF-A

In order to identify new genes potentially involved in angiogenesis process, we performed a deep-sequencing transcriptome analysis of HUVECs stimulated with 50 ng/mL recombinant VEGF-A for six hours, after growing the cells in complete endothelial growth medium (EGM) deprived of VEGF-A for 24 h. The analysis was performed calculating the average of stimulated and non-stimulated samples, each one was performed in triplicate.

**Figure 1:**
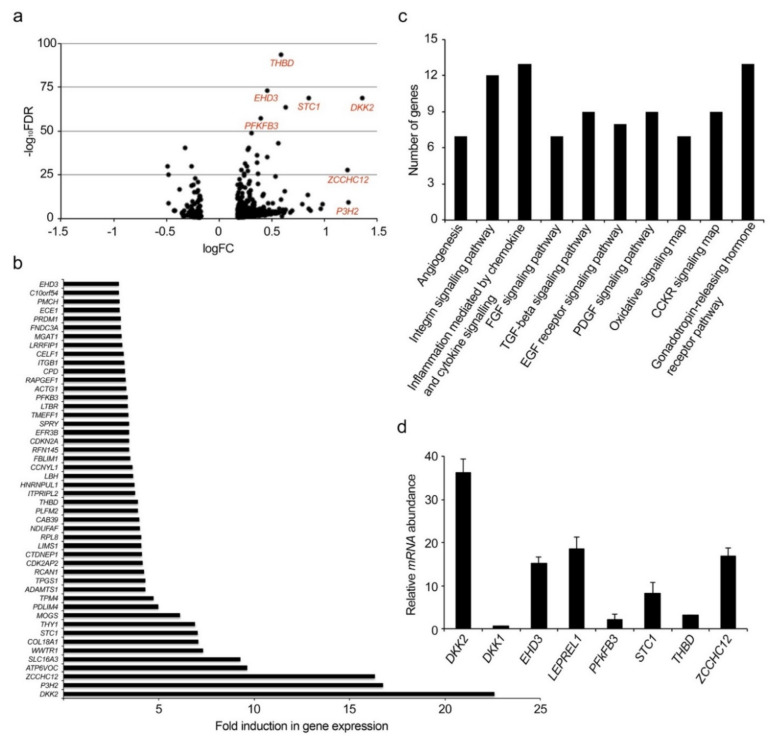
Analysis and validation of RNAseq of human umbilical vein endothelial cells (HUVECs) stimulated with VEGF-A. (**a**) Volcano plot of significant differentially expressed up- and downregulated genes based on the logFC and −log_10_FDR. In red the validated upregulated genes are shown. (**b**) Expression level of 48 genes upregulated >3-fold by VEGF-A showed as fold induction compared to not induced HUVECs with, a *p*-value <0.1. Analysis of gene expression was obtained using the edgeR package from the BioConductor collection. (**c**) Panther pathways analysis, in the graph the enriched pathway that hits seven or more genes are shown. (**d**) Validation of expression of some genes regulated by VEGF-A in HUVECs with respect to vehicle treated cells. VEGF-A upregulates *DKK2, EHD3, P3H2, PFKFB3, STC1, THBD,* and *ZCCHC12* and downregulates *DKK1* mRNAs, as evaluated by qRT-PCR. Data are presented as the mean ± SEM of three independent experiments performed in triplicate.

Expression level of each RefSeq annotated gene was performed with the HTSeq program and differential expression analysis was evaluated by using the edgeR package from the BioConductor collection. We selected as differentially expressed those genes showing a ±1.5-fold change and a corrected *p*-value smaller than 0.1. On the basis of these criteria, we found that 459 (343 up-regulated and 116 down-regulated genes) of around 8000 expressed genes were significantly regulated by VEGF-A, as summarized in the volcano plot shown in Figure 1a, while in Figure 1b the 48 genes upregulated more than threefold are listed. The differentially expressed genes in VEGF-A treated cells were subjected to a bioinformatics analysis using PANTHER (Protein ANalysis THrough Evolutionary Relationships, www.pantherdb.org (Access Date: 27 April 2020)) to perform functional pathway analysis. In Figure 1c are reported the most abundant and significant enriched pathways with at least 7 genes involved: angiogenesis (P00005; 7 genes), integrin signaling pathways (P00034; 12 genes), inflammation mediated by chemokine and cytokine signaling pathway (P00031; 13 genes), FGF signaling pathway (P00021; 7 genes), TGF-beta signaling pathway (P00052; 9 genes), EGF receptor signaling pathway (P00018; 8 genes); PDGF signaling pathway (P00047; 9 genes), oxidative stress response (P00046; 7 genes), CCKR signaling map (P06959; 9 genes), gonadotropin-releasing hormone receptor pathway (P06664; 13 genes). Noteworthy, all these enriched pathways are strictly related to the angiogenesis process.

To validate the results of the deep-sequencing analysis, we examined the expression of 20 genes chosen among the most up-regulated and those that have never been associated to angiogenesis process, by using qRT-PCR as a quantitative and independent method. The analysis validated almost 80% of chosen genes. In Figure 1d is shown the validation of seven upregulated transcripts: dickkopf WNT signaling pathway inhibitor 2 (DKK2) [31], stanniocalcin 1 (STC1) [32], thrombomodulin (THBD), and 6-phosphofructo-2-kinase/fructose-2,6-biphosphatase 3 (PFKFB3) [33], already associated to VEGF-A modulation and/or angiogenesis process, and *P3H2* [17], zinc finger CCHC-type containing 12 (ZCCHC12) [34] and EH domain containing 3 (EHD3) [35], which have never been directly correlated with angiogenesis, to date. We also validated dickkopf WNT signaling pathway inhibitor 1 (DKK1) that was downregulated by VEGF-A in our analysis, as previously demonstrated [31]. *P3H2* was the second most upregulated gene with an increase of 16.7-fold as compared to non-stimulated cells.

Collectively these data indicate that the experimental conditions chosen for RNAseq experiment have represented a valuable approach to identify new genes modulated by VEGF-A possibly associated to angiogenesis process.

### 2.2. VEGF-A Stimulates P3H2 Expression in Human Endothelial Cells through VEGFR-2/p38 Signaling

**Figure 2:**
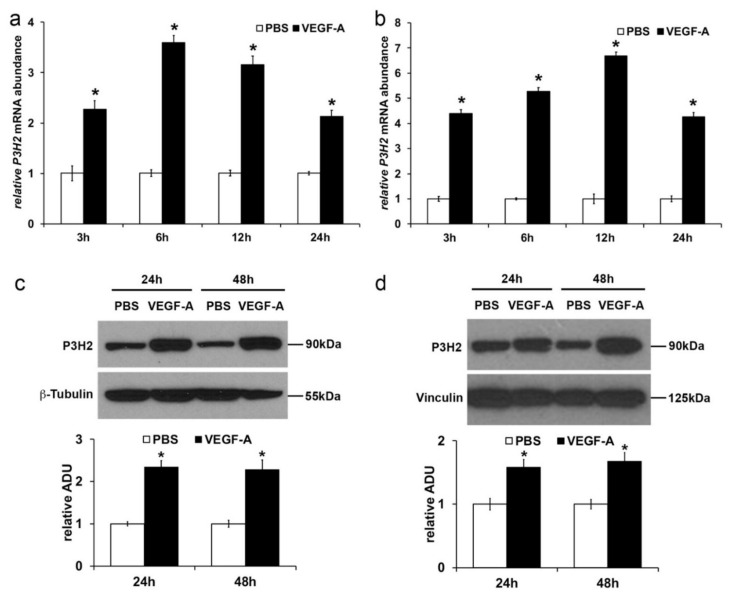
VEGF-A induces *P3H2* expression on HUVECs and HDMVECs. VEGF-A increases *P3H2* mRNA (black bars) in HUVECs (**a**) and HDMVECs (**b**) with respect to phosphate buffered saline (PBS) treated cells (white bars) as evaluated by qRT-PCR (* *p* < 0.005 compared to PBS). Data are presented as the mean ± SEM of three independent experiments performed in triplicate. Representative pictures of Western blot analysis and densitometric analysis of the level of *P3H2* protein in HUVECs (**c**) and HDMVECs (**d**) after VEGF-A stimulation. *P3H2* abundance were normalized against β-Tubulin or Vinculin, respectively. Data are presented as the mean ± SEM of three independent experiments (* *p* < 0.05 compared to PBS).

Time-dependent expression of *P3H2* was evaluated by qRT-PCR in HUVEC and HDMVEC primary human endothelial cells after stimulation with VEGF-A. In both cell lines, VEGF-A was able to induce *P3H2* transcript upregulation over time up to 24 h compared to non-stimulated cells (Figure 2a,b). In line with these data, to the overexpression of *P3H2* mRNA corresponded an increase of *P3H2* protein after 24 and 48 h from VEGF-A stimulation, as assessed by Western blot analysis in HUVECs (Figure 2c) and HDMVECs (Figure 2d).

**Figure 3:**
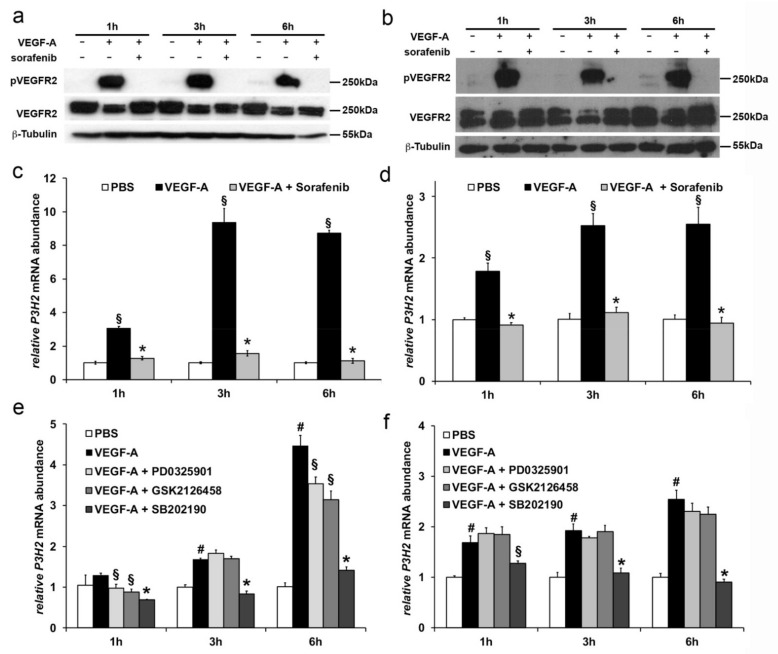
VEGF-A induces *P3H2* expression through VEGFR-2/p38 MAPK signaling cascade. Sorafenib inhibits the VEGF-A/VEGFR-2 signaling cascade. Western blot analysis of pVEGFR-2 and VEGFR-2 in protein extracts of HUVECs (**a**) and HDMVECs (**b**) stimulated with VEGF-A in the presence or absence of Sorafenib. β-Tubulin antibody was used for normalization. VEGF-A induces up-regulation of *P3H2* mRNA in HUVECs (**c**) and HDMVECs (**d**), which is fully inhibited by Sorafenib pre-treatment as evaluated by qRT-PCR. Data are presented as the mean ± SEM of three independent experiments performed in triplicate (HUVECs: § *p* < 0.001 compared to PBS; * *p* < 0.005 compared to VEGF-A; HDMVECs: § *p* < 0.006 compared to PBS; * *p* < 0.005 compared to VEGF-A). Up-regulation of *P3H2* mRNA induced by VEGF-A is blocked over time in HUVECs (**e**) and HDMVECs (**f**) by p38 MAPK inhibitor pre-treatment (SB202190), but not by pre-treatment with MEK1/2 (PD0325901) and PI3K (GSK2126458) inhibitors, as evaluated by qRT-PCR. Data are presented as the mean ± SEM of three independent experiments performed in triplicate. (HUVECs: **#** *p* < 0.001 compared to PBS; * *p* < 0.001 and § *p* < 0.05 compared to VEGF-A; HDMVECs: **#** *p* < 0.005 compared to PBS; § *p* < 0.05 and * *p* < 0.005 compared to VEGF-A). After 6 h of stimulation with VEGF-A, MEK1/2 (PD0325901), and PI3K (GSK2126458) inhibitors slightly affected *P3H2* expression in HUVECs.

VEGFR-2 is the main receptor activated by VEGF-A on endothelial cells stimulating the angiogenic switch [2]. To evaluate if its activation is involved in *P3H2* upregulation, endothelial cells were stimulated with VEGF-A in presence or absence of Sorafenib, a multitarget inhibitor of TK receptors able to block VEGFR-2 but not the other receptor recognized by VEGF-A and expressed on endothelial cells, the VEGFR-1 [36]. As shown in Figure 3a,b both in HUVECs and HDMVECs, VEGF-A induced a robust VEGFR-2 phosphorylation compared to non-stimulated cells, which resulted abolished by the pretreatment with Sorafenib. Therefore, we carried out qRT-PCR analysis to evaluate the level of expression of *P3H2* transcript, whose increase induced by VEGF-A was fully blocked by pretreatment with Sorafenib (Figure 3c,d).

Upon VEGF-A binding, VEGFR-2 is able to activate several mediators of signal transduction. In order to evaluate which of them is involved in *P3H2* upregulation, HUVECs and HDMVECs were stimulated with VEGF-A after pre-incubation with MEK1/2 (PD0325901), PI3K (GSK2126458), and p38 MAPK (SB202190) specific inhibitors. The p38 MAPK inhibitor strongly reduced the *P3H2* overexpression in both HUVECs and HDMVECs, already after 1 h of VEGF-A exposure. In HUVECs, after 6 h of stimulation, the MEK1/2 and PI3K inhibitors induced a slight reduction of *P3H2* upregulation compared to the VEGF-A-stimulated cells (Figure 3e,f).

Collectively, these results clearly indicate that the *P3H2* transcription is positively modulated by VEGF-A in endothelial cells through the activation of VEGFR-2/p38 MAPK signaling cascade.

### 2.3. Modulation of P3H2 Expression Affects Angiogenic Properties of Endothelial Cells

In order to evaluate the effects of *P3H2* expression on angiogenic properties of HUVECs, proliferation, migration, and capillary sprouting assays were performed after gain- and loss-of-function experiments.

**Figure 4:**
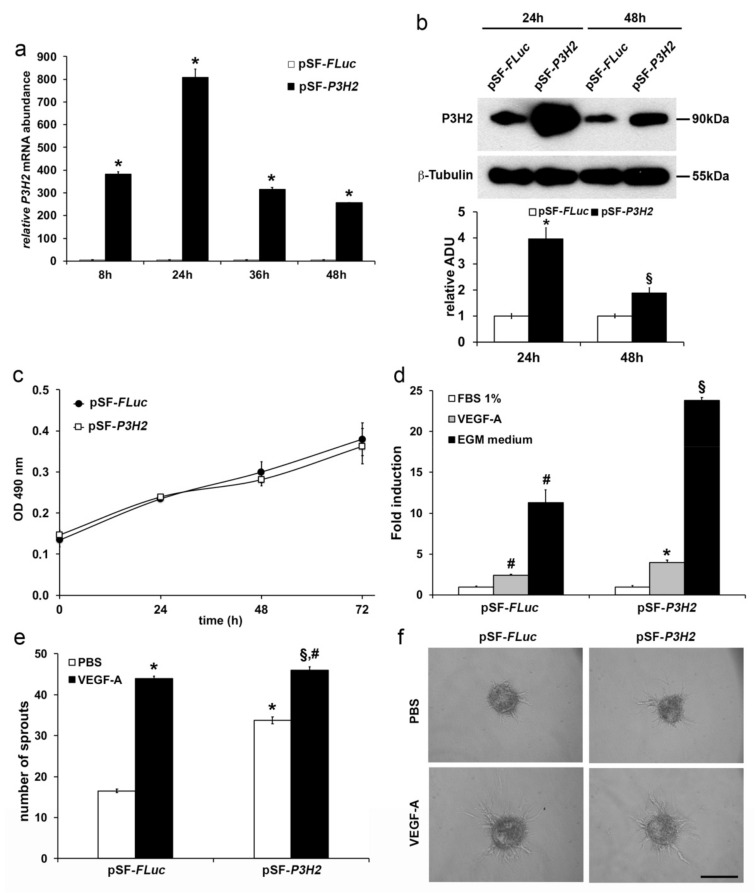
*P3H2* overexpression increases angiogenic properties of ECs. Efficiency of *P3H2* overexpression in HUVECs after transfection with pSF-*P3H2* as evaluated (**a**) by qRT-PCR (data are presented as the mean ± SEM of three independent experiments performed in triplicate, * *p* < 0.0001 compared to pSF-FLuc transfected cells) and (**b**) by Western blot (representative pictures of Western blot analysis and densitometric analysis of *P3H2* normalized against β-Tubulin; data are presented as the mean ± SEM of three independent experiments, * *p* < 0.005 and § *p* < 0.05 compared to pSF-*Fluc*). (**c**) Proliferation of pSF-*Fluc* and pSF-*P3H2* transfected HUVECs was evaluated at indicated time using the CellTiter Aqueous One Cell Proliferation Assay (Promega). Data are presented as the mean ± SEM of three independent experiments performed in triplicate. (**d**) *P3H2* overexpression achieved by pSF-*P3H2* transfection increases HUVECs migration after stimulation with either VEGF-A and endothelial growth medium (EGM) medium. Data are presented as the mean ± SEM of three independent experiments performed in triplicate. # *p* < 0.005 compared to FBS 1% pSF-FLuc transfected cells; * *p* < 0.01 and § *p* < 0.002, compared to pSF-FLuc transfected cells stimulated with VEGF-A or EGM, respectively. (**e**) HUVEC spheroids transfected with pSF-*P3H2* or pSF-FLuc embedded in fibrin gel were incubated with VEGF-A or with vehicle (PBS). Formation of radially growing sprouts was evaluated after 24 h of incubation. N = 25 spheroids per group. Data are presented as the mean ± SEM of two independent experiments. (* *p* < 0.0001 and § *p* < 0.05 compared to pSF-FLuc spheroids treated with PBS or VEGF-A, respectively; # *p* < 0.001 compared to pSF-*P3H2* spheroids treated with PBS) (**f**) Representative pictures of growing sprouts from HUVECs transfected spheroids stimulated with VEGF-A or vehicle (PBS). Scale bar 100 µm.

Gain-of-function of *P3H2*—The overexpression of *P3H2* was achieved transfecting HUVECs with an expression plasmid coding *P3H2* cDNA (pSF-*P3H2*) and the transfection efficiency was evaluated in terms of *P3H2* mRNA and protein abundance by qRT-PCR and Western blot analyses. As shown in Figure 4a,b, both mRNA and protein showed a peak of *P3H2* overexpression after 24 h from transfection, as compared to cells transfected with a control plasmid carrying the cDNA of *Firefly Luciferase* (pSF-*FLuc*). While *P3H2* overexpression did not affect proliferation (Figure 4c), the ability of HUVECs transfected with pSF-*P3H2* (HUVECs-pSF-*P3H2*) to migrate toward a stimulatory signal, such as VEGF-A or complete endothelial growth (EGM) medium, was doubled compared to HUVECs transfected with pSF-*FLuc* (HUVECs-pSF-*FLuc*) (Figure 4d and Figure S1).

Moreover, capillaries sprouting assays [37] were performed stimulating with VEGF-A endothelial spheroids generated with HUVECs-pSF-*P3H2* or HUVEC-pSF-*FLuc*. As expected, VEGF-A was able to strongly stimulate capillary sprouting from HUVEC-pSF-*FLuc* spheroids compared to spheroids treated with vehicle (phosphate buffered saline (PBS)). Surprisingly, the simple overexpression of *P3H2* induced per se an increase of the basal capillary sprouting, and the stimulation with VEGF-A significantly improved the capillary sprouting from HUVECs-pSF-*P3H2* spheroids, as compared to VEGF-A-induced capillary sprouting from HUVECs-pSF-*FLuc* spheroids (Figure 4e,f).

Collectively, these data show that the overexpression of *P3H2* in HUVECs does not affect proliferation but positively modulates their response to migratory signals, it increases the ability to form capillaries from HUVEC endothelial spheroids, an effect that results amplified in response to VEGF-A stimulus.

**Figure 5:**
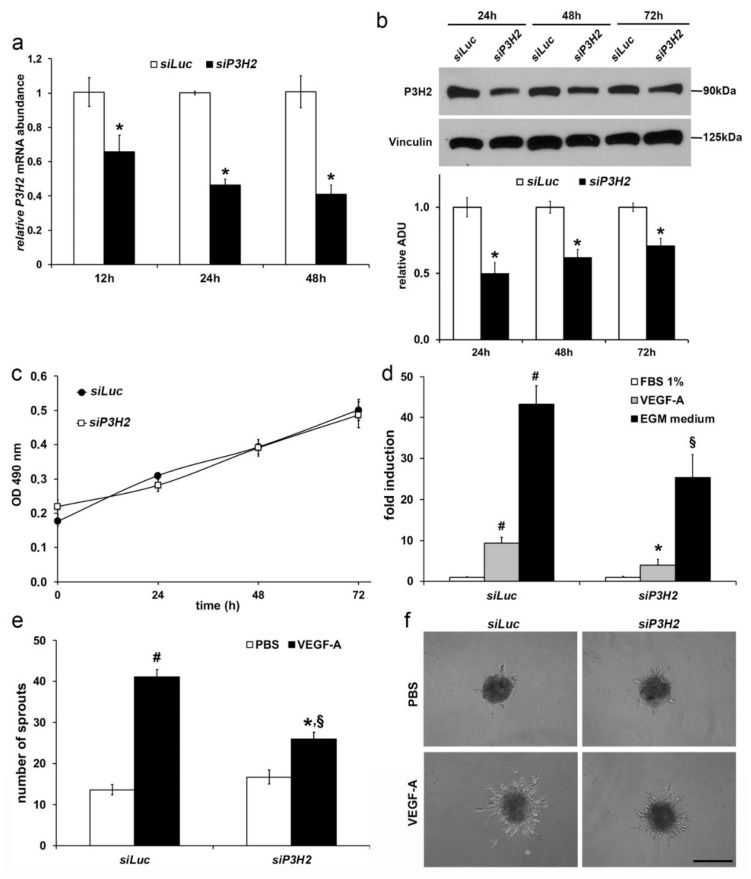
*P3H2* downregulation decreases angiogenic properties of ECs. *siP3H2* transfection in HUVECs is able to efficiently knockdown target mRNA and decreases protein amount over time compared to cells transfected with *siLuc,* as evaluated by (**a**) qRT-PCR (data are presented as the mean ± SEM of three independent experiments performed in triplicate, * *p* < 0.05 compared to *siLuc*) and (**b**) by Western blot (representative pictures of Western blot analysis and densitometric analysis of *P3H2* normalized against Vinculin; data are presented as the mean ± SEM of three independent experiments, * *p* < 0.05 compared to si*Luc*). (**c**) Proliferation of *siLuc* and *siP3H2* transfected HUVECs was evaluated at indicated time using the CellTiter Aqueous One Cell Proliferation Assay (Promega). Data are presented as the mean ± SEM of three independent experiments performed in triplicate. (**d**) *P3H2* knockdown achieved by *siP3H2* transfection decreases HUVECs migration after stimulation with either VEGF-A or EGM medium. Data are presented as the mean ± SEM of three independent experiments performed in triplicate. # *p* < 0.005 compared to FBS 1% *siLuc* transfected cells; * *p* < 0.02 and * *p* < 0.005, compared to *siLuc* transfected cells stimulated with VEGF-A or EGM, respectively. (**e**) HUVEC spheroids transfected with *siP3H2* and *siLuc* embedded in fibrin gel were incubated with VEGF-A or with vehicle (PBS). Formation of radially growing sprouts was evaluated after 24 h of incubation. N = 25 spheroids per group. Data are presented as the mean ± SEM of two independent experiments. (# *p* < 0.0001 compared to *siLuc* spheroids treated with PBS; * *p* < 0.0001 compared to *siLuc* spheroids treated with VEGF-A; § *p* < 0.001 compared to *siP3H2* spheroids treated with PBS). (**f**) Representative pictures of growing sprouts from HUVEC transfected spheroids stimulated with VEGF-A or vehicle (PBS). Scale bar 100 µm.

Loss-of-function of *P3H2*—The knockdown of *P3H2* expression was obtained by transfecting HUVECs with a sequence-specific siRNA (*siP3H2*). As control, a siRNA against *Firefly Luciferase* (*siLuc*) was used. qRT-PCR analysis showed a reduction of about 35% of *P3H2* mRNA after 12 h from transfection that reached the 60% after 48 h, compared to control (Figure 5a). In parallel, Western blot analysis showed a reduction of *P3H2* protein (−50% after 24 h) that was maintained up to 72 h (−29%) (Figure 5b). As observed with the *P3H2* gain-of-function, HUVECs proliferation was not affected by *P3H2* knockdown (Figure 5c). The ability of HUVECs to migrate in response to VEGF-A or EGM medium was inhibited of about 50% by *P3H2* knockdown (Figure 5d and Figure S1). In capillaries sprouting assay, the reduction of *P3H2* determined a reduction of 35% of the ability of HUVEC spheroids to respond to VEGF-A, compared to control (Figure 5e,f).

Collectively, these data mirror the results obtained in gain-of-function experiments confirming a direct role of *P3H2* in in vitro angiogenic properties of endothelial cells.

### 2.4. Overexpression of P3H2 Induces Collagen IV Rearrangement In Vitro

**Figure 6:**
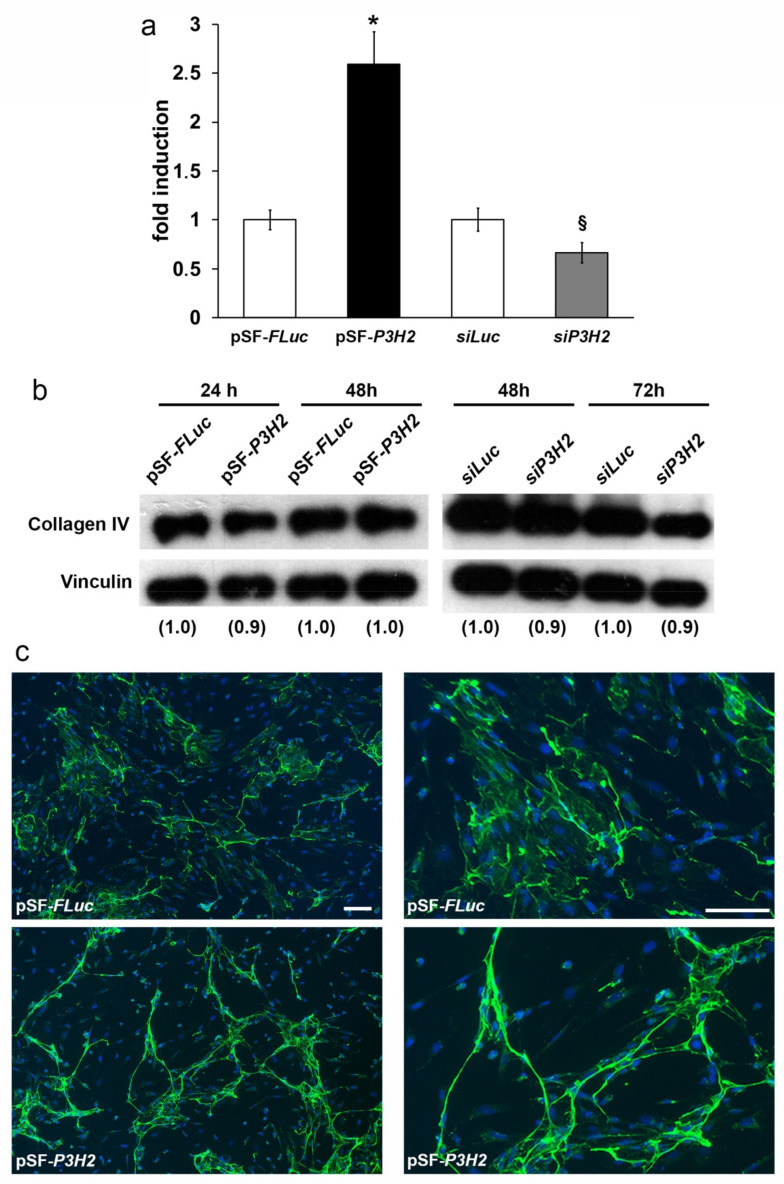
*P3H2* induces Collagen IV rearrangement. *P3H2* modulates the total amount of hydroxyproline. (**a**) Integrated density of Hyps staining on HUVECs transfected with pSF-*P3H2* or *siP3H2*. The ratio positive area of Hyps staining with respect to the number of nuclei stained with 4′,6-diamidino-2-phenylindole (DAPI) was determined using the ImageJ software in 15 optical fields per sample. Data are expressed as fold induction with respect to pSF-*Fluc* (* *p* < 0.001) and *siLuc* (§ *p* < 0.05) transfected cells. (**b**) Collagen IV amount in HUVECs transfected with pSF-*P3H2* or *siP3H2*, and as control with pSF-*FLuc* or *siLuc* respectively, was evaluated by Western blot over time. Densitometric values normalized against Vinculin are shown in parentheses. (**c**) Representative images of immunostaining of type IV Collagen on HUVECs transfected with pSF-*P3H2* and pSF-*Fluc*, as control. Nuclei are counterstained with DAPI (blue). Scale bars: 100 μm.

Since *P3H2* catalyzes the hydroxylation of Pro in 3Hyp in collagens [22,23], we decided to verify whether the gain- and the loss-of-function of *P3H2* in HUVECs determine a detectable change in the total amount of Hyps. Immunofluorescence analyses performed in HUVECs with an antibody that recognizes all forms of Hyps showed a significant increase of total level of Hyps after 24 h from pSF-*P3H2* transfection and a significant decrease of Hyps after 72 h from *siP3H2* transfection, compared to cells transfected with pSF-*FLuc* and *siLuc*, respectively (Figure 6a).

Considering that the main substrate of *P3H2* is the Collagen IV, we also investigated the consequence of *P3H2* expression variations on Collagen IV in HUVECs transfected with *pSF-P3H2* or *siP3H2*. No substantial change of Collagen IV protein abundance was observed by Western blot analysis in both cases (Figure 6b).

Interestingly, immunofluorescence analysis showed that the *P3H2* overexpression induced an impressive rearrangement of Collagen IV, which appeared more condensed, forming structures resembling the capillary-like tube formation along which HUVECs were aligned, with respect to control in which cells and Collagen IV appear more dispersed (Figure 6c).

These results indicate that an increase of 3-Hyp residues induces a reorganization of Collagen IV mimicking a pro-angiogenic microenvironment, at least in vitro.

### 2.5. P3H2 Modulates In Vivo Pathological Angiogenesis

In order to evaluate the role of *P3H2* in in vivo angiogenesis, we used the laser-induced choroid neovascularization (CNV) model that recapitulates the wet form of age-related macular degeneration [38]. In this model, the upregulation of VEGF-A after laser damage drives the formation of new pathological vessels [39].

**Figure 7:**
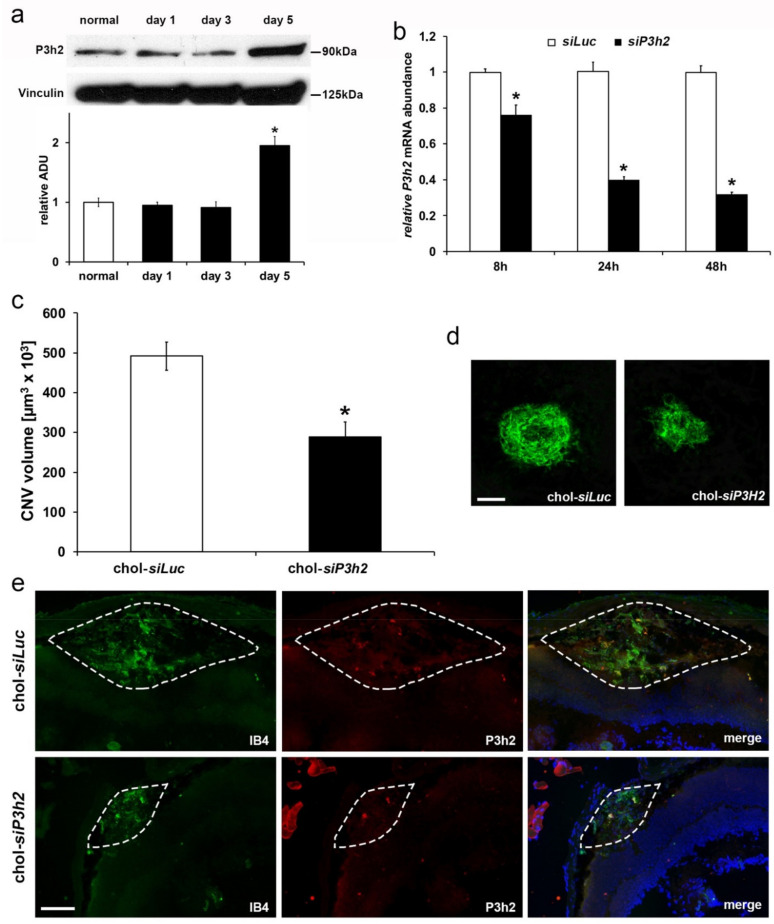
*P3H2* knockdown reduces laser-induced choroid neovascularization (CNV). (**a**) *P3H2* is up-regulated in RPE/choroid tissues during laser-induced CNV as evaluated by Western blot. Densitometric analysis of *P3H2* normalized against Vinculin; data are presented as the mean ± SEM of three independent experiments, * *p* < 0.005 compared to normal. (**b**) *siP3H2* transfection in mRPE is able to efficiently knockdown its target mRNA as compared to cells transfected with *siLuc,* as evaluated by qRT-PCR. (**c**) chol-*siP3H2* or chol-*siLuc* were intravitreally injected in C57Bl6/J mice before CNV induction. CNV volume was measured by Isolectin B4 staining of RPE-choroid flat mounts after 7 days from laser-induced damage. Data are presented as the mean ± SEM of two independent experiments (*n* = 10 mice per group; * *p* = 0.001 versus chol-*siLuc)*. (**d**) Representative pictures of CNV. Scale bar: 100 µm. (**e**) *P3H2* staining (red), that decreases in chol-*siP3H2* compared to chol-*siLuc* injected eyes, co-localizes with IB4 (green) in the area of CNV lesion after 5 days from laser-induced damage. Nuclei were stained blue by DAPI. CNV lesion are highlighted with dashed line. No specific immunofluorescence was detected with isotype control IgGs (Figure S2). Scale bar: 100 µm.

First of all, we investigated whether *P3H2* resulted upregulated in vivo in retinal pigment epithelium (RPE) cells/choroid tissues isolated from C57/Bl6 mice after laser-induced lesions, in a time dependent experiment. The levels of *P3H2* protein remained unchanged after one and three days but doubled after five days from laser burns with respect to non-lasered tissues, as evaluated by Western blot analysis (Figure 7a). Then, we showed by qRT-PCR analysis that in primary mouse retinal pigment epithelial (mRPE) cells the sequence-specific siRNA designed against mouse *P3H2* (*siP3H2*) was effectively able to knockdown *P3H2* mRNA, compared to the control siRNA *siLuc* (Figure 7b).

In order to verify whether the inhibition of *P3H2* expression could affect the formation of new vessels in vivo, we intravitreally injected C57Bl6/J mice with the chol-*siP3H2*, which was shorter than 21 nucleotides in length to prevent TLR3 activation and conjugated to cholesterol to enable cell permeation [40]. The injection was accomplished immediately after the delivery of laser lesions and the extent of CNV volume was measured after seven days by immunofluorescence labeling with Isolectin B4 (IB4). Interestingly, the eyes injected with chol-*siP3H2* showed a significant reduction of the neovascularization of about 40% compared to chol-*siLuc* injected eyes (Figure 7c,d). In addition, immunofluorescence analyses of retinal sections at level of CNV lesions with IB4 and anti-*P3H2* antibody showed that these two signals co-localized, indicating that *P3H2* is expressed by the choroid endothelial cells (Figure 7e and Figure S2). Furthermore, in agreement with CNV volume results, chol-*siP3H2* injected eyes showed a reduced *P3H2* expression together with a restricted CNV area compared to chol-*siLuc* injected eyes (Figure 7e).

**Figure 8:**
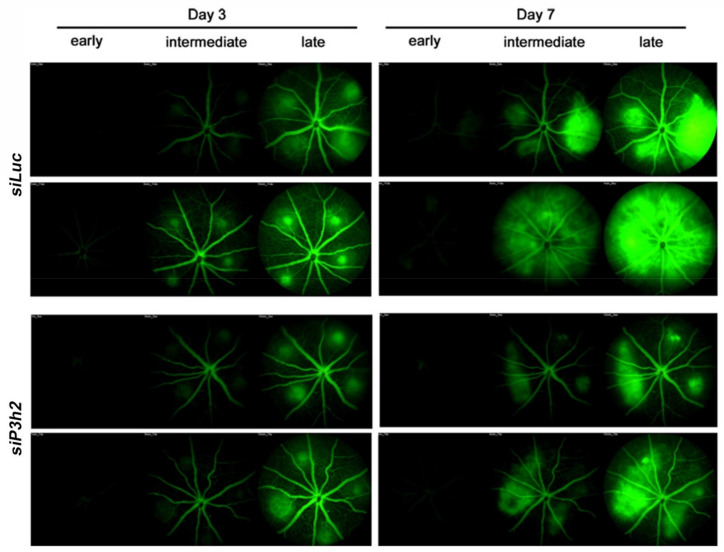
*P3H2* knockdown reduces vascular leakage. Representative images of fundus fluorescein angiography were acquired at three different times (early 1 min, intermediate 5 min, late 15 min) following intraperitoneal delivery of fluorescein after 3 and 7 days from laser damage.

Moreover, we also performed a qualitative fundus fluorescein angiography (FFA) at days 3 and 7 after laser injury. This analysis showed a slight fluorescein leakage in eyes injected with chol-*siP3H2* at day 3, that become much more evident after 7 days, as compared to chol-*siLuc* injected eyes (Figure 8).

Overall these data demonstrate that *P3H2* is directly involved in the modulation of new vessel formation in the pathological context of experimental CNV.

## 3. Discussion

Anti-angiogenic therapy has become a routine approach in the treatment of certain tumors and of ocular diseases in which pathological neovascularization is involved. Since angiogenesis is involved in a further high number of pathological states, such as rheumatoid arthritis, osteoarthritis, hemangioma and vascular malformation, obesity, psoriasis, new anti-angiogenic therapies are highly demanded, possibly developing additional or alternative strategies to anti-VEGF therapy whose effect is limiting in certain conditions [5]. In order to identify alternative targets, we have performed a deep sequencing analysis on primary endothelial cells (ECs) after VEGF-A stimulation in vitro, not on starved cells but on cells cultured to maintain the best condition for their survival and propagation. This novel approach turned out to be interesting because other than genes known to be modulated by VEGF-A and already associated to angiogenesis process, it allowed us to identify new genes upregulated by VEGF-A which function has never been associated to angiogenic properties of ECs, so far.

Here, we focus our attention on *P3H2* gene, and we demonstrate that its expression is positively modulated by VEGF-A in two human primary endothelial cell lines and that this upregulation occurs mainly via VEGFR-2/p38MAPK signaling pathway, which is able to induce the activation of several transcription factors and biological process [2]. p38MAPK is one the main mediator of the migratory ability of endothelial cells. Indeed, the activation of p38MAPK and focal adhesion kinase is essential for the recruitment of the actin-binding vinculin to initiate endothelial cell migration [41,42].

Therefore, the data obtained on the migratory ability of ECs observed after gain- and loss-of-function of *P3H2* are consistent with that on the signaling pathway.

Moreover, we have observed that the simple overexpression of *P3H2* confers to endothelial spheroids the ability to activate capillary sprouting showing that an increase of *P3H2* activity is necessary and sufficient to stimulate the formation of new vessels, other than better respond to pro-angiogenic stimuli.

Collagen type IV is the main substrate of *P3H2* catalytic activity and is the more abundant component of the basement membrane (BM). BM is a multifunctional support that, depending on the concentration of its molecular components, is able to mediate several biological processes including organogenesis, tissue repair, cellular behavior, and angiogenesis [43,44]. One of the first step of angiogenesis in vivo, consists into degradation of BM [45,46]. Indeed, the loss of contact among ECs and BM induces a specialized phenotype in ECs, known as endothelial tip cells, which confers the ability to detect and migrate versus the angiogenic stimulus [47]. In parallel, ECs capability to synthetize and deposit Collagen IV represents a central event in blood vessel formation since it is indispensable for vascular survival and maturation in vivo [29,30,48].

Moreover, post-translational modifications of collagens are crucial events for the structural and functional features of these molecules, such as triple helix assembly and stability, intermolecular cross-linking, and strength of fibrils [19,49]. The most important modifications are represented by the hydroxylation of lysine and proline residues driven by enzymes belonging to the family of 2-oxo-glutarate-dependent dioxygenases. Lysine hydroxylation is a modification essential for the formation of intermolecular cross-links, whereas proline hydroxylation, in particular proline 4-hydroxylation, is essential for the establishment of secondary and tertiary structure of collagen [50,51,52] and for the thermal stability of the newly synthesized triple-helical collagens [19].

The functional role of proline 3-hydroxylation catalyzed by P3Hs is still under debate. Indeed, whereas it has been suggested that 3-Hyp may introduce lower stability within the triple helix that may be required for the assembly of some supramolecular structures in the BM [53], it was also reported that the destabilization is small when the presence of 3-Hyp occurs in the natural Xaa position [54] of Xaa-4Hyp-Gly collagen triplet, since the crystal structure maintains the prototypical triple-helix structure and the absence of unfavorable steric interactions [55].

Our immunofluorescence analysis in HUVECs overexpressing *P3H2* shows a more condensed status of Collagen IV, suggesting that to an increase of proline-3-hydroxylation activity corresponds a reorganization of Collagen IV from a dispersed to more organized structure that is also accompanied by an alignment of the cells along the Collagen IV bundles, so towards an evident pro-angiogenic status.

Corroborating the data obtained in vitro, *P3H2* knockdown is able to significantly reduce pathological angiogenesis in the model of laser-induced CNV. It is well established that experimental CNV is driven by upregulation of VEGF-A [39]. Therefore, the upregulation of *P3H2* observed in RPE cells/choroid tissue after 5 days from laser-induced damage suggests that *P3H2* expression is also stimulated by VEGF-A in vivo. Furthermore, to the reduced CNV volume was also associated a consequent reduction of vascular leakage, as confirmed by the fundus fluorescein angiography.

These in vivo results, together with the spontaneous capillary sprouting from endothelial spheroids overexpressing *P3H2*, strongly support the conclusion of a direct involvement of *P3H2* in angiogenic process.

Previous studies of mutations or knockout of *P3H2* and COL4 genes have never been associated to phenotypes directly linked to the angiogenic process. Interestingly both genes are involved in eye diseases. The loss-of-function of *P3H2* generated by two different single mutations has been associated to non-syndromic severe myopia with early-onset cataract and variable expressivity of vitreoretinal degeneration and subluxated lens [56,57]. Similar alterations were observed in a knockout model of *P3H2*. These mice were viable and fertile and the analysis of adult eye tissues demonstrated that the absence of 3Hyp in Collagen I from sclera and in Collagen IV from lens capsule, determined structural abnormalities that are compatible with the phenotypes observed in patients [58]. On the other hand, another study describing *P3H2* knockout reported embryonic lethality at day 8.5 due to abnormal maternal blood clotting triggered by glycoprotein VI binding to 3Hyp-deficient type IV collagen of the embryo [59]. Due to these opposite results, we are generating in house a *P3H2* conditional knock out model to overcome these conflictual data.

Type IV collagens, are encoded by three pairs of paralogous genes, collagen type IV alpha 1 (COL4A1) through COL4A6. COL4A1, and COL4A2 are highly conserved across species and dominant-negative mutations in these genes are pleiotropic and contribute to a broad spectrum of disorders including myopathy, glaucoma, and cerebrovascular disease [60]. Col4a1 and Col4a2 mutant mice model mirror several types of human disease such as anterior segment ocular dysgenesis, glomerulopathy and spontaneous intracerebral hemorrhage [61,62,63]. In the retina, Col4a1 and Col4a2 are mainly present in basement membranes of the choriocapillaries vasculature [64,65] and Col4a1 mutation causes highly penetrant and progressive retinopathy that is secondary to vascular defects [66].

Therefore, loss-of-function of *P3H2*, which alters post-translational collagens modifications, has been associated to some eye defects in which structural abnormalities are present. Our data also highlight the importance of this enzyme and of the post-translational collagens modifications in the context of ocular neovascular diseases.

Overall our data point to an unforeseen mechanism of the biological function of *P3H2*. Although a direct relationship among Collagen IV and angiogenic process has been described, here we demonstrate that *P3H2*, through its activity on Collagen IV, is a molecular player directly involved in the modulation of new vessels formation. Consequently, *P3H2* may be considered as a new target for the development of possible therapeutic approaches for ocular neovascular diseases as well as for all other diseases in which pathological angiogenesis is involved.

## 4. Materials and Methods

### 4.1. Cell Culture

Human umbilical vein endothelial cells (HUVECs, Lonza, Basel, Switzerland) were cultured in endothelial basal medium (EBM-2) supplemented with 2% fetal bovine serum (FBS) and endothelial growth factors (basic Fibroblast Growth Factor, Insulin-like Growth Factor 1, Epidermal Growth Factor, heparin, hydrocortisone, ascorbic acid and VEGF-A; EGM-2 bullet kit, Lonza). HUVECs at passages 4–5 were used for all the experiments. Human Primary Dermal Microvascular Endothelial Cells from Neonatal Foreskin (HDMVECs, ATCC, Manassas, VA, USA), were cultured in Vascular Cell Basal Medium supplemented with endothelial growth factors (Microvascular Endothelial Cell Growth Kit-VEGF, ATCC); HDMVECs at passages 4–5 were used for all the experiments. 6 to 8weeks-old C57Bl6/J were used to isolate primary mouse RPE cells (mRPE). Eyeball cleaned from fat and extra tissues was digested with 1:1 mixture of 0.8mg/mL collagenase and 4% dispase. After the removal of cornea and anterior segment the “eye cup” was flattened with four incisions and digested 10min at 37 °C with 2% dispase. Then, the RPE were scraped from sclera in 12 well plate (1 eye/well) with 20% FBS DMEM with antibiotics by rubbing the cup against the bottom of the well with a pipette tip. Cells were grown until confluence and then passed. mRPE cells at passages 4–5 were used for all the experiments and were cultured in DMEM (Dulbecco’s Minimal Essential Medium, Euroclone, Pero (MI), Italy) containing 20% FBS and standard antibiotics. All cell lines were grown at 37 °C in a humidified environment containing 5% CO_2_.

### 4.2. RNAseq

HUVECs were stimulated for six hours with 50 ng/mL VEGF-A (R&D Systems), after growing them for 24 h with EGM without VEGF-A. PBS treated cells were used as negative control. Total RNA was isolated by TRIZOL (Thermo Scientific) according to the manufacturer’s protocol. RNA quality was determined using a NanoDrop spectrophotometer (Thermo Scientific, Waltham, MA, USA). RNA quality was determined using Agilent bioanalyzer 2100 (Agilent Technologies, Santa Clara, CA, USA). The analysis showed clear, defined 28S and 18S rRNA peaks, an indication of high-quality preparation. RNA extracted from stimulated and non-stimulated HUVEC (each sample in triplicate) has been submitted to the Genomics Core Facility at EMBL (Heidelberg, Germany) for RNAseq on an Illumina HiSeq2000 platform using the TruSeq v2 protocol. Expression level of each RefSeq annotated gene was performed with the HTSeq program and differential expression analysis by using the edgeR package from the BioConductor collection. For each comparison we selected as differentially expressed those genes showing a fold change higher than 1.5 and a corrected *p*-value smaller than 0.1. The differentially expressed genes in VEGF-A-treated cells were subjected to a bioinformatics analysis using PANTHER (Protein ANalysis THrough Evolutionary Relationships, www.pantherdb.org) (Access Date: 27 April 2020) to perform functional pathway analysis. RNAseq datasets were submitted to EMBL-EBI with accession number E-MTAB-9337.

### 4.3. VEGF-A Stimulation and VEGFR-2 Signaling Inhibition

HUVECs were seeded at 30,000 cells/cm^2^ and starved in EBM-2 1% FBS for 16 h before the induction with 50 ng/mL VEGF-A (R&D System). Where indicated, Sorafenib (1 µm, Selleck, Houston, TX, USA), MEK1/2 (0.1 µm PD0325901, Merk Millipore, Billerica, MA, USA), PI3K (0.1 µm GSK21264580, GlaxoSmithKline, Brentford, United Kingdom) and p38 MAPK (100 µm SB202190, SIGMA, St Louis, MO, USA) specific inhibitors was added 1 h before VEGF-A treatment.

### 4.4. Quantitative Real-Time PCR

Total RNA was isolated using Trizol reagent (Invitrogen, Carlsbad, CA, USA), DNase treated and reverse transcribed (QuantiTect, QIAGEN, Hilden, Germany). The RT products (cDNA) were amplified by real-time quantitative PCR on CFX96TM Real Time PCR Detection Systems (BioRad, Hercules, CA, USA) with SYBR green Master Mix. Relative expression was determined by the 2^− ΔΔCt^ method using human *18S* or mouse *Actin* as an internal control. Each point was done in triplicate. Oligonucleotide primers specific human *DKK1* (forward 5′-CATCAGACTGTGCCTCAGGA-3′ and reverse 5′-TATCCGGCAAGACAGACCTT-3′) human *DKK2* (forward 5′-GAGATCGAAACCACGGTCAT-3′ and reverse 5′-GAAATGACGAGCACAGCAAA-3′), human *EHD3* (forward 5′-CCCACCACAGACTCCTTCAT-3′ and reverse 5′-GCTCTCCAGCACAGGGTTAG-3′), human *P3H2* (forward 5′-GTGCAACTGTCCTGAAAGCA-3′ and reverse 5′-TCGGCAGACCATGTGTGTAT-3′), human *PFKFB3* (forward 5′-CACTTGCATTACCGTCCCTG-3′ and reverse 5′-ACTCTTCCGACCTTCCCAAG-3′), human *STC1* (forward 5′-CACACCCACGAGCTGACTTC-3′ and reverse 5′-TCTCCCTGGTTATGCACTCTCA-3′), human *THBD* (forward 5′-CAGAGAGGCCTTTTGGAATGTG-3′ and reverse 5′-TTCTAACCAGCTCCCATGGG-3′), human *ZCCHC12* (forward 5′-GGATACCAGCACATTGGAGGG-3′ and reverse 5′-TATACCACTTTCACAAAGAATAAAGCTG-3′), human *18S* (forward 5′-CGCAGCTAGGAATAATGGAATAGG-3′ and reverse 5′-GCCTCAGTTCCGAAAACCAA-3′), mouse *P3H2* (forward 5′-TTGGTGATGGATACCGAGGG-3′ and reverse 5′-TCCACAATCTTCCGAGCCTT-3′) and mouse *Actin* (forward 5′-CGGTTCCGATGCCCTGAGGC-3′ and reverse 5′-GAGCAATGCCTGGGTACATGGTGG-3′). The qPCR cycling conditions were 50 °C for 2 min, 95 °C for 10 min, followed by 40 cycles of a two-step amplification program (95 °C for 15 s and 58 °C for 1 min).

### 4.5. Expression Vector

The vector pSF-CMV-PGK-*FLuc* (pSF-*FLuc*) was purchased from SIGMA. The ORF of human *P3H2* was excised from pEZ-Lv205 vector (GeneCopoeia, Rockville, MD, USA) with PmeI and XhoI sites and cloned in EcoRV and XhoI sites of pSF-*FLuc* vector to generate pSF-*P3H2* expression vector.

### 4.6. Transient Transfection

HUVECs plated at 30,000 or 10,000 cells/cm^2^ were transfected with pSF-*P3H2*, pSF-*Fluc* or with *siP3H2* (5′-ACUUCGAACAAGCCUUAdTdT-3′) and mRPE plated at 10,000 with *siP3H2* (5′-GCAUUUGUCAAACGUCAdTdT-3′). As control *siLuc* (5′-UAAGGCUAUGAAGAGAUdTdT-3′) were transfected. Lipofectamine 2000 (Invitrogen) was used for transfection according to the manufacturer’s instructions.

### 4.7. Western Blot

Western blot experiments were performed following standard procedures. First, 50–100 µg of total protein extracts were run on 10% SDS-polyacrylamide gel and transferred to a PVDF membrane (Millipore, Burlington Middlesex County, MA, USA) and incubated with antibodies against human *P3H2* (SIGMA, 1:1000). Protein loading was assessed using antibody anti β-Tubulin (Santa Cruz Biotechnology, Dallas, TX, USA, 1:2000) or anti-Vinculin (Cell Signaling, Danvers, MA, USA 1:10,000). The secondary antibodies were from DAKO, Santa Clara, CA, USA, (1:10,000). The signals were visualized by chemiluminescence using ECL substrate (Advansta, Menlo Park, CA, USA).

### 4.8. Cell Proliferation

Proliferation of HUVECs in gain- and loss-of-function experiments was evaluated each 24 h up to 72 h using the CellTiter Aqueous One Cell Proliferation Assay (Promega, Madison, WI, USA) following the manufacturer’s procedure.

### 4.9. Cell Migration

After 8 h from transfection HUVECs were starved for 16 h and then 35,000 cells were seeded into the upper chamber of a 24-multiwell insert system with 5 μm pore size polycarbonate filter (Corning, NY, USA) while 600 μL of EBM-2 containing or not VEGF-A (50 ng/mL) and EGM medium were added to the lower chamber. After 48 h, the cells on the top of the filter were removed by gentle swabbing and the remaining cells on the bottom side of the filter were stained with DAPI. Images were recorded on Nikon fluorescence microscope and single cells counted using ImageJ (NIH, Bethesda, MD, USA).

### 4.10. Spheroids Sprouting Assay

After 8 h from transfection HUVECs were suspended at a density of 4000 cells/mL in EBM culture medium containing 0.4% Methylcellulose and 10% FBS. 800 cells were seeded into non-adherent round bottom 96-well plates (Corning, NY, USA) and cultured overnight at 37 °C. The spheroids were harvested by gently pipetting and centrifuged at 300× *g* for 15 min. The spheroids were then suspended and embedded in fibrin in the presence of 10 µg/mL aprotinin to prevent the dissolution of the substrate. The sprouting was stimulated with VEGF-A, or with vehicle (PBS) as control. After 16 h at 37 °C, sprouts were counted and phase-contrast images were captured with an inverted microscope (Leica).

### 4.11. Immunofluorescence Analyses

HUVECs were fixed with 4% PFA for 10 min, permeabilized in 0.1% Triton-X-100 in PBS for 10 min and blocked one hour in 1% BSA, 20% Goat Serum in PBS 0.4% Triton-X-100 (PBS-T). After blocking, cells were exposed to primary antibodies against Collagen IV (Santa Cruz Biotechnology, Dallas, TX, USA, 1:50) or an anti-Hydroxyproline antibody able to recognize all forms of Hyps (Abcam 1:100, cat.*n*. Ab37067, Cambridge, United Kingdom) in blocking solution, and incubated 16 h at 4 °C in a humid chamber. After three washes in PBS-T, Alexa-Fluor conjugated antibodies diluted 1:400 were incubated for 30 min. Slides were mounted with Vectashield with DAPI (Vector Laboratories, Burlingame, CA, USA) to counterstained nuclei. Images were recorded on Nikon fluorescence microscope.

### 4.12. Animals

Six- to eight-week-old C57Bl6/J male mice were purchased from Charles River (Milan, Italy). For all procedures, anesthesia was achieved by intraperitoneal injection of 100 mg/kg ketamine hydrochloride and 10 mg/kg xylazine. Mice were treated in accordance with European directives no. 2010/63/UE and Italian directives D.L. 26/2014, and were approved by the Italian Ministry of Health (authorization no. 695/2015-PR of 17 July 2015).

### 4.13. Choroid Neovascularization Model

Laser photocoagulation was performed on 6–8 weeks old C57Bl6/J mice (*n* = 10 per group), with a Micron IV apparatus (Phoenix Research Labs, Pleasanton, USA). The presence of a massive sub-retinal hemorrhage after laser induction of CNV determined the exclusion of relative eyes from the analysis. Chol-*siP3H2* and chol-*siLuc* were purchased from Sigma-Aldrich (St Louis, MO, USA) and were dissolved in phosphate buffered saline (PBS) to obtain a concentration of 1 µg/µL. Immediately after the laser injury, 1 µL of chol-*siP3H2* were intravitreally injected in C57Bl6/J mice with a microsyringe (Hamilton, Manitowoc, WI, USA) carrying a 33-gauge needle. As control, 1 μL of chol-*siLuc* was delivered to the contralateral eyes. Seven days after laser injury, eyes were enucleated, the eye-cups isolated and stained with 0.7% FITC-conjugated Griffonia simplicifolia Isolectin B4 (Vector Laboratories, Burlingame, CA, USA). Afterwards, retinae were removed and RPE-choroid were flat mounted by four incisions under dissecting microscope and then mounted with Vectashield. Leica DM6000 fluorescent microscope was used to visualize CNV and to obtain horizontal optical sections at every 1-μm step from the surface to the deepest focal plane. The CNV volume was obtained by the sum of the whole fluorescent area of each optical section by using ImageJ software.

### 4.14. Retinal Immunostaining

Five days after laser-induced injury, eyes were enucleated, snap-frozen in OCT, and cryosectioned. 10 μm-thick sections were fixed 20 min with PFA 4%. Slides were then washed three times in PBS and blocked 1 h in 10% normal goat serum (NGS), 1% bovine serum albumin (BSA), 0.1% Triton-X-100 and 0.05% Tween-20 with and then stained overnight at 4 °C with rabbit anti-*P3H2* (SIGMA, 1:50) and with 0.7% FITC-conjugated Griffonia simplicifolia Isolectin B4 (Vector Laboratories, Burlingame, CA, USA) to identify CNV lesion. The following day, sections were washed three times and incubated with Alexa-Fluor 564-conjugated donkey anti-rabbit antibody (1:250; Jackson Antibodies) for 1 h at RT. Sections were then mounted with Vectashield with DAPI (Vector Laboratories, Burlingame, CA, USA). Rabbit IgG isotype labeling instead of primary antibody was used as a negative control for all experiments. Images were acquired on Nikon fluorescence microscope.

### 4.15. Fundus Fluorescein Angiography

The Fundus Fluorescein Angiography (FFA) was performed after pupil dilatation with topical application of 1% tropicamide. Before imaging, Gel 4000 was applied on the cornea to prevent dehydration and to eliminate the cornea’s refractive power. For FFA 2% fluorescein sodium (Akorn) was injected intraperitoneally. Images were captured at three different time points: 1 (early), 5 (intermediate), and 15 (late) min using GFP filters (excitation, 482 nm, emission, 536 nm) of Micron IV apparatus (Phoenix Research Labs).

### 4.16. Statistical Analyses

Results are expressed as mean ± SEM, with *p* values < 0.05 considered statistically significant. Differences among groups were compared by the Student’s *t* test.

## Data Availability

RNA-seq datasets were submitted to EMBL-EBI with accession number E-MTAB-9337.

## Supplementary Materials

The following are available online at https://www.mdpi.com/article/10.3390/ijms22083896/s1, Figure S1: Representative images of cell migration experiments. After migration, HUVECs were fixed on filters and nuclei were stained with DAPI.

## Abbreviations

AMD	age-related macular degeneration
BM	basement membrane
CNV	choroid neovascularization
DKK1	dickkopf WNT signaling pathway inhibitor 1
DKK2	dickkopf WNT signaling pathway inhibitor 2
ECs	endothelial cells
EHD3	EH-domain containing 3
HMEC	human dermal microvascular endothelial cells
HUVEC	human umbilical vein endothelial cells
LEPREL1	Leucine proline-enriched proteoglycan like 1
LV	lentivirus
mRPE	mouse retinal pigment epithelial cells
P3Hs	prolyl 3-hydroxylases
*P3H2*	prolyl 3-hydroxylase 2
PFKFB3	6-phosphofructo-2-kinase/fructose-2,6-biphosphatase 3
STC1	stanniocalcin 1
VEGF-A	vascular endothelial growth factor A
VEGFR1	VEGF receptor 1
VEGFR2	VEGF receptor 2
ZCCHC12	Zinc Finger CCHC-Type Containing 12
3Hyp	3-hydroxyl-l-proline
4Hyp	4-hydroxyl-l-proline
