# Supplementary material for: Prolyl 3-Hydroxylase 2 Is a Molecular Player of Angiogenesis
Source: Int J Mol Sci. 2021 Apr 9;22(8):3896. doi: 10.3390/ijms22083896 (PMC8069486; doi:10.3390/ijms22083896)
Supplement: Supplementary file 1 [file ijms-22-03896-s001.pdf]

# Supplementary Figures

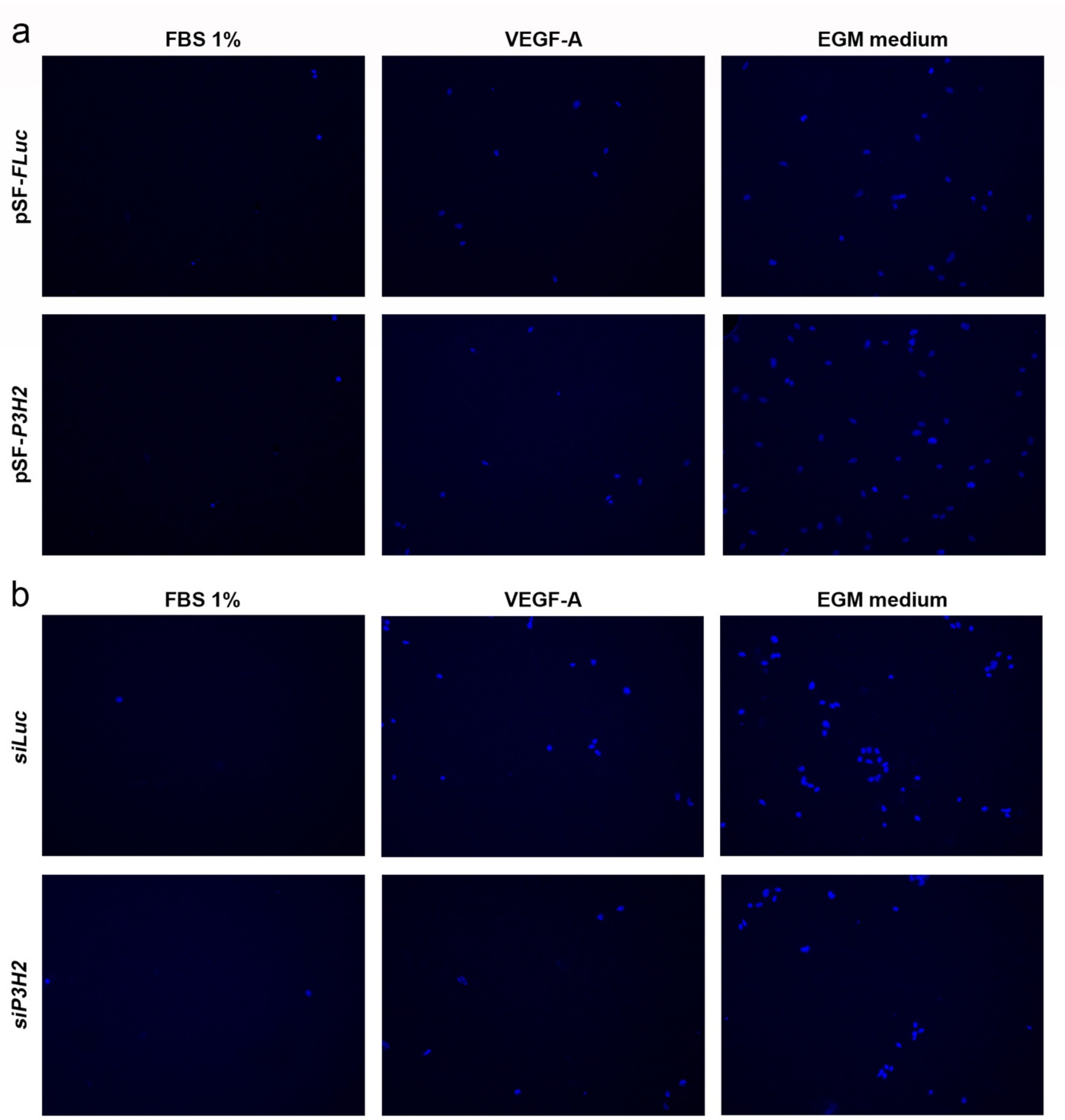


**Figure S1.** Representative images of cell migration experiments. After migration, HUVECs were fixed on filters and nuclei were stained with DAPI. HUVECs transfected with (**a**) pSF-*P3H2*, or pSF-*Fluc* as control, or (**b**) with *siP3H2*, or *siLuc* as control. Migration was stimulated with VEGF-A or complete EGM medium, and with FBS 1% as control.

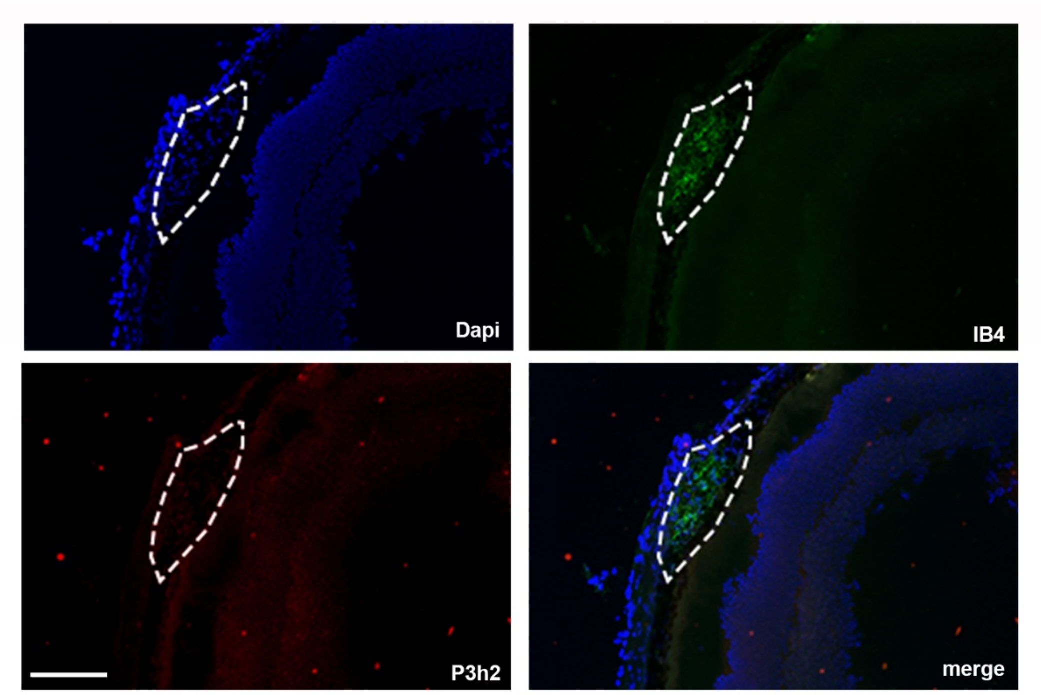


**Figure S2.** Staining of retinal section with: isotype control IgGs (red), IB4 (green), DAPI (blue), after 5 days from laser-induced damage. CNV lesion are highlighted with dashed line. Scale bar: 100μm.
